# Somatic Deletion in Exon 10 of Aryl Hydrocarbon Receptor Gene in Human GH-Secreting Pituitary Tumors

**DOI:** 10.3389/fendo.2020.591039

**Published:** 2020-11-12

**Authors:** Agnese Re, Francesco Ferraù, Concetta Cafiero, Federica Spagnolo, Valeria Barresi, Daniela Petronilla Romeo, Marta Ragonese, Claudio Grassi, Alfredo Pontecorvi, Antonella Farsetti, Salvatore Cannavò

**Affiliations:** ^1^Institute for Systems Analysis and Computer Science “A. Ruberti” (IASI), National Research Council (CNR), Rome, Italy; ^2^Department of Human Pathology of Adulthood and Childhood, University of Messina, Messina, Italy; ^3^Endocrine Unit, University Hospital G. Martino, Messina, Italy; ^4^Medical Oncology, S.G. Moscati Hospital, Taranto, Italy; ^5^Department of Diagnostic and Public Health, Section of Pathology, University of Verona, Verona, Italy; ^6^Dipartimento di Neuroscienze, Università Cattolica del Sacro Cuore, Rome, Italy; ^7^Fondazione Policlinico Universitario A. Gemelli IRCCS, Rome, Italy; ^8^Dipartimento di Medicina e Chirurgia Traslazionale, Università Cattolica del Sacro Cuore, Rome, Italy

**Keywords:** pituitary adenoma, acromegaly, aryl hydrocarbon receptor, somatic deletion, sequencing analysis

## Abstract

**Objective/Purpose:**

The aryl hydrocarbon receptor (AHR) pathway plays a critical role in the biology of Growth Hormone (GH)-secreting pituitary tumor (somatotropinoma). Germline rs2066853 *AHR* variant was found to be more frequent among acromegaly patients and associated with a more severe disease with larger invasive somatropinoma, and with resistance to somatostatin analogs treatment in patients living in polluted areas. However, no somatic changes in *AHR* gene have been reported so far in acromegaly patients. On that basis, the aim of the study was to assess at the somatic level the *AHR* gene status encompassing exon 10 region, also because of the high rate of variants found in this genomic region.

**Methods:**

A cohort of 13 patients aged 20–76 years with biochemical, clinical and histological diagnosis of somatotropinoma was studied. DNA and RNA from pituitary tumor histological samples have been extracted and analyzed by PCR and direct sequencing for *AHR* gene variants, and compared with corresponding patients’ germline DNA as well as normal pituitary tissue as reference control.

**Results:**

A degenerated letter codes in the region corresponding to *AHR* exon 10 (c.1239-c.2056) was detected in somatotropinomas-derived DNA but not in that of matched germline and pituitary normal tissue. By multiple PCR and sequencing analysis, we observed amplification only before codon 1246 and after codon 1254, confirming the presence of a tumor-restricted somatic deletion in the 5’ upstream region of *AHR* exon 10. Analysis of PCR-amplified cDNA revealed a wildtype sequence of exon 9 and 10 in normal pituitary tissue, and a wildtype sequence of exon 9 and 10 up to codon 1246 and no sequence after the deletion region (c.1246-c.1254) in 6 out of 9 tumor samples. Patients carrying the germline rs2066853 *AHR* variant showed no somatic LOH at the corresponding genetic locus.

**Conclusion:**

This is the first demonstration of a recurrent somatic deletion in the exon 10 of the *AHR* gene in somatotropinomas. The functional impact of this genetic finding needs to be clarified.

## Introduction

Among pituitary tumors, the growth hormone (GH)-secreting ones (somatotropinomas) account for about 15%–20% of cases, causing gigantism in children/adolescents while in adults leads to the clinical picture of acromegaly characterized by overgrowth of bone and cartilages, and cardiovascular, metabolic, respiratory and neoplastic complications (1–3). Somatotropinoma tumorigenesis and pathophysiology is only partially understood, although several efforts have been made to identify underlying genetic and epigenetic abnormalities (4). In terms of genetic background, the most frequently somatic pathogenic events in somatotropinomas (around 35% of cases) are gain-of-function mutations in the stimulatory guanine nucleotide (GTP) binding protein alpha (Gas) encoded by the GNAS gene, with consequent constitutive cAMP pathway activation (3, 5). On the other hand, next generation sequencing approach has revealed few relevant somatic alterations in these tumors such as USP8, GNAS, USP48, and BRAF (5). In this context, it is worth mentioning that the aryl hydrocarbon receptor (AHR)-interacting protein (AIP) has been proven critical in somatotropinoma physiopathology and clinical expression (6, 7). Indeed, germline inactivating AIP mutations can predispose to GH-secreting pituitary tumor formation and associate with a more aggressive clinical phenotype (6). AHR, an orphan receptor involved in xenobiotics detoxification and in many other biological activities, is the intracellular AIP key partner. Structurally, AHR is composed of several active domains: a N-terminal bHLH domain responsible for DNA binding, two median PAS domains that serve as ligand binding region, and a glutamine-rich transcriptional activation domain (TAD) located at the C-terminal (8, 9). TAD is responsible for protein-protein interactions with co-regulators and transcription factors (10, 11). In addition, in this domain reside most of the known polymorphisms. AHR appears to act as tumor suppressor in GH-secreting pituitary adenomas (PA) (12). Our research group recently showed that, in patients with acromegaly living in highly polluted areas, germline *AHR* rs2066853 variant, associate with a more severe clinical and biochemical phenotype, with larger and more invasive somatotropinomas and resistance to somatostatin analogs treatment (13–15). These data are in line with the *in vitro* findings suggesting that a dysfunctional AHR and/or endocrine disruptors exposure might affect pituitary tumor biology (7, 12, 16, 17). Nevertheless, despite clear evidence of a potential role of AHR in somatotropinomas pathophysiology and clinical relevance, somatic *AHR* gene alterations in acromegaly patients have not been reported.

These observations prompted us to assess the *AHR* gene status at somatic level in somatotropinomas, including the potential loss of heterozygosity at the locus of the rs2066853 variant.

## Materials and Methods

### Human Pituitary Adenoma Tissue Specimens

The study cohort consisted of 13 patients (8 women and 5 men) aged 20–76 years with biochemical, clinical and histopathological diagnosis of GH-secreting pituitary adenoma, referred to the Endocrine Unit of the University Hospital of Messina. Six sections from formalin-fixed paraffin-embedded (FFPE) of each patient and relative germline DNA were obtained. Postoperative acromegaly remission was observed in eight patients while the remaining five showed disease persistence or recurrence. Germline *AHR* gene status was known for all of them: five harboured the *AHR* rs2066853 variant. Other clinical characteristics of the patient cohort are in **Table 1**.

**Table 1:** Clinical, radiological, and pathological characteristics of patients and tumors.

Case N.	Sex	Age	Dimension (mm)	Knosp grade	GH ng/ml^a^	IGF-1 ULN^a^	Remission	Adenoma type	Ki67 (%)	p53 (%)	Germline rs 2066853	Germline AIP mutations
#1	M	63	43	3b	3,03	4,6	No	somatotroph	10	15	Yes	No
#2	M	23	20	1	21,8	4,12	Yes	somatotroph	4	1	No	No
#3	F	20	18	2	20,9	2,3	Yes	somatotroph	5	2	Yes	No
#4	F	76	15	2	10,9	6,9	No	somatotroph	1	0	Yes	No
#5	M	57	11	1	2	4,14	Yes	somatotroph	<1	0	Yes	No
#6	M	67	13	1	0,6	1,3	Yes	somatotroph	2	N/A	No	No
#7	F	29	18	1	28	1,61	No	somatotroph	N/A	N/A	No	No
#8	M	57	22,5	2	12	3,35	No	somatotroph	1	0	No	No
#9	F	36	15	1	7	1,97	Yes	somatotroph	N/A	N/A	No	No
#10	F	56	25	2	19	2,44	No	somatotroph	N/A	N/A	No	No
#11	F	44	16	1	6,7	11	No	somatotroph	N/A	N/A	Yes	No
#12	F	38	25	3a	31,8	2,9	No	somatotroph	N/A	N/A	No	No
#13	F	35	15	1	64	1,9	No	somatotroph	<1	0	No	No

N/A, not available; a, at diagnosis.

The study was approved by the Ethical Committee of the University Hospital of Messina (Protocol n. 111/16, as of 8/11/2016) and performed in accordance with the ethical standards stated in the Declaration of Helsinki. Patients approved the experimental approach and signed the consensus form prior to be enrolled in the study.

### Genomic DNA Extraction and Sequencing

A pathologist reviewed the haematoxylin and eosin slides staining to identify tumor *vs* normal tissue and tumor areas were subsequently dissected from an unstained FFPE section by manual microdissection. Genomic DNA was extracted using the QIAamp DNA FFPE Tissue Kit (QIAGEN) according to the manufacturer protocol. Five 10-μm thick FFPE sections were used for each patient. Germline DNA was extracted from fresh blood samples as described in Cannavò S. et al. (14). All samples were normalized to the same DNA concentration.

Polymerase Chain Reaction (PCR) amplification was performed in a total volume of 20 μl, containing 10 μl of PCR Master Mix 2X (ProMEGA Corporation) and 1 μl of each primer at concentration of 10 pmol/μl. The reaction was carried in a Thermocycler PCR System 9700 (MWG) with an initial denaturation step at 95°C for 3 min and then 36 cycles at 95°C for 45 s, 55–60°C for 45 s, 72°C for 45 s, followed by 5 min of final extension at 72°C. PCR primers as described in Cannavò et al. (13) (exon 2, 5, and 10) or designed using Genamics Expression DNA Sequence Analysis Software and the in Silico-PCR tool provided by the UCSC Genome Browser (http://rohsdb.cmb.usc.edu/GBshape/cgi-bin/hgPcr). PCR products were purified using ExoSTAR 1 Step (EuroClone), directly sequenced on both strands using BigDyeTerminator V3.1 and subsequently resolved on an ABI3130 Genetic Analyzer (Applied Biosystems, Foster City, USA). NM_001621.5 was used as reference sequence of *AHR* gene. Identified variations were confirmed in a new PCR and sequencing reaction.

Primers for exon 10 DNA genotyping were as follows:

#A: chr7:17338740+17339068 (329bp) Intron 9-10Fw 5’- GTAGGGATGTAACCTGACC-3’ and Exon 10_83bp 5’- CATACAACACAGCTTCTCCAGTG-3’
#B: chr7:17339084+17339404 (321bp) Exon 10_98bpFw 5’- TTCCTGCCATAATGGATCCC-3’ and Exon 10_Rev 5’- GACCTCCAGCAAATGAGTT-3’
#C: chr7:17339322+17339650 (329bp) Exon 10_336bpFw 5’- TACTGCACCGATGGGAAATG-3’ and Exon 10_10-666bp_Rev 5’- AGTTCAGAGCCAAGGACTG-3’
#D: chr7:17339632+17339880 (249bp) Exon 10_C Fw 5’- CAGTCCTTGGCTCTGAACT-3’ and Exon 10_B Rev 5’- GAACTCTTGACTGATCCCAT-3’
chr7:17338986+17339118 (133bp) Exon 10_Del Fw 5’ AGATGAGGAAGGAACAGAGCATT-3’ and Del_Rev 5’- AGTCCTTAGTGGTAAGGGATCCA-3’

### RNA Extraction and Sequencing

Total RNA from tumor tissue section identified by pathologist was extracted and purified using a Qiagen’s RNeasy FFPE Kit. The quantity and quality of the RNA were checked by Nanodrop (E260, E260/E280 ratio, spectrum 220–320 nm). cDNA was produced according to standard protocols. cDNA genotyping was performed using internal primers for exon 9-10 (Exon 9Fw: 5’- GACTGGAGAAAGTGGCATGATAGTT-3’; Exon 10Rev pre-deletion: 5’- CATACAACACAGCTTCTCCAGTG-3’ or Exon 10Rev post-deletion 5’- GACCTCCAGCAAATGAGTT -3’). PCR reaction and sequencing analysis were performed as for genomic DNA.

### *In Silico* Analysis

Sequence data obtained from the analysis of exon 10 of the *AHR* gene were inspected using Sanger Sequencing and Fragment Analysis Software SeqScape of Applied Biosystems (ThermoFisher Scientific). The reference mRNA sequence is NM_001621.5 and variants are indicated starting from the first nucleotide of the coding sequence (+1). The reference protein sequence is NP_001612.1. The variants within the sequence of exon 10 of the *AHR* gene (c.1661G>A chr7:g.17339486G>A (hg38) p.Arg554Lys; c.1246_1254del chr7:g.17339071 (hg38) p.416/417/418) were searched in gene-specific databases, such as Ensembl Genome Browser (www.ensembl.org), the Leiden Open Variation database - LOVD (https://databases.lovd.nl/shared/genes/AHR) and the NCBI ClinVar database (https://www.ncbi.nlm.nih.gov/clinvar). In addition, we searched the general population databases, namely the NCBI Database of Short Genetic Variation – dbSNP (https://www.ncbi.nlm.nih.gov/snp) and the Genome Aggregation Database - GnomAD (https://gnomad.broadinstitute.org/). Analysis of these variants has been performed also with specific database for somatic mutations such as COSMIC and the Catalogue Of Somatic Mutations In Cancer.

## Results

### Characterization of Somatic *AHR* Gene Variants in GH-Secreting Pituitary Tumors

**Figure 1:**
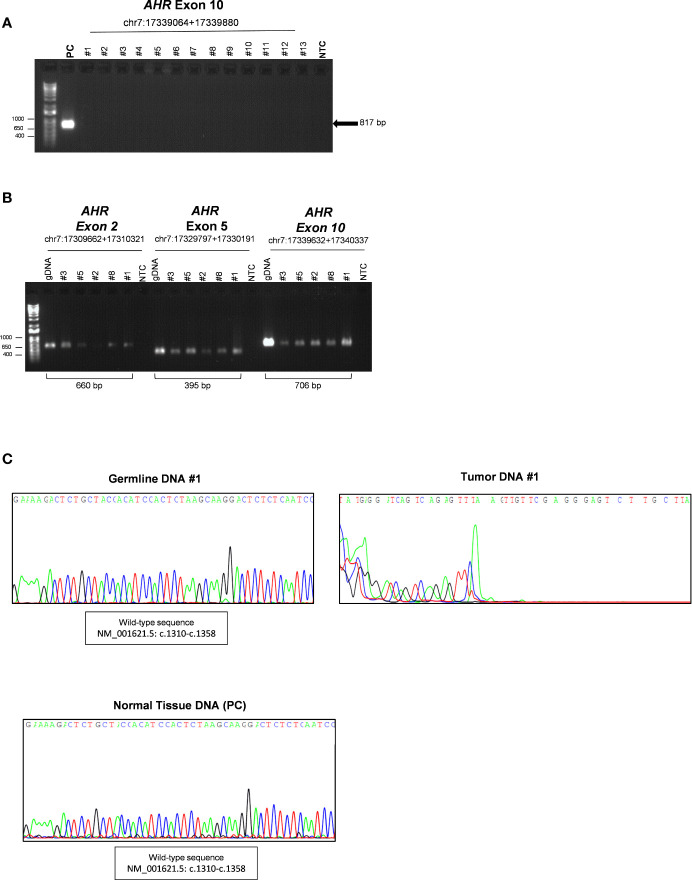
Characterization of somatic aryl hydrocarbon receptor (*AHR*) mutation in Growth Hormone (GH)-secreting pituitary tumors. **(A)** Image of a DNA 1% agarose gel. The leftmost lane contains DNA ladder (1kb plus). Lane 2 shows 817 base pair amplified PCR product of *AHR* exon 10 (chr7:17339064+17339880; c.1239-c.2056) of healthy human pituitary sample use as positive control (PC). The subsequent lanes contain a PCR reaction of DNA extract from tumor tissue of patient with (lanes 3- 7) or without (lanes 8-15) germline polymorphism rs2066853 show lack of detectable amplification of *AHR* exon 10. Lane 16 shows negative control (NTC; Non-template control). **(B)** Image of a DNA 1% agarose gel of representative PCR of germline and tumor DNA for *AHR* exons 2 (chr7:17309662+17310321), five (chr7:17329797+17330191) and 10 downstream region (chr7:17339632+17340337; (c.1808-intron 10-11). The leftmost lane contains DNA ladder (1kb plus). Lanes 2, 9 and 16 show PCR product of germline DNA (gDNA) as reference control. Lanes 3-7, 10-14 and 17-21 shown PCR product of tumor DNA of patient #3, #5, #2, #8 and #1. Lanes 8, 15 and 22 show negative control without DNA template (NTC; Non-template control). **(C)** Representative sequence analysis of *AHR* exon 10. Electropherograms show the sequence of codon site c. 1310-c.1358 in germline (left panel) and tumor tissue DNA from the patient #1 (right panel), and healthy pituitary sample (lower panel), use as positive control (PC).

Both germline and somatic DNA was first analyzed by PCR using primers validated for sequencing analysis of germline DNA (gDNA) (chr7:17339632+17340337, c.1239-c.2056) to analyze exon 10 of *AHR* gene. Surprisingly, as shown in **Figure 1A**, no PCR products of *AHR* exon 10 (c.1239-c.2056) was detected in tumor samples compared to pituitary normal tissue, used as reference control. PCR amplification of other exons of *AHR* (2 and 5, chr7:17349286+17349945 and chr7:17369421+17369815, respectively) and a downstream 5’ region of exon 10 (chr7:17379256+17379961, c.1808-intron 10-11) was detected in tumor tissue and germline DNAs, suggesting the presence of a putative variant in the upstream exonic region 10 (**Figure 1B**). As expected, sequence analysis showed a degenerated letter codes in tumor DNA (**Figure 1C**, right panel, and **Figure S1**) as compared to DNA from blood of the same patients (**Figure 1C**, left panel, and **Figure S2**) and healthy pituitary tissue (**Figure 1C**, lower panel, and **Figure S3**), suggesting a putative upstream deletion in the *AHR* exon 10 only in tumor tissue.

All these data confirmed that the lack of detectable sequence amplification is restricted to the 5’ upstream region of *AHR* exon 10 and present exclusively in tumor tissue.

### Validation of *AHR* Somatic Deletion

**Figure 2:**
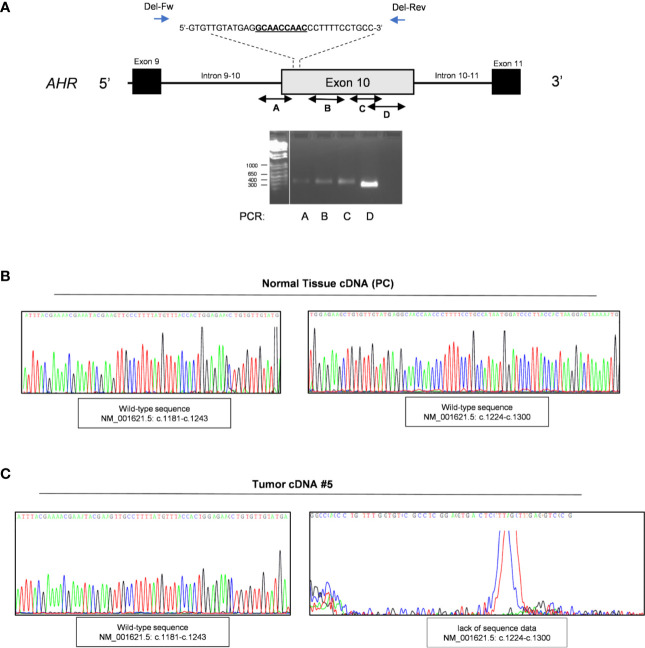
Validation of in frame somatic deletion on tumor DNA. **(A)** Schematic image of the human aryl hydrocarbon receptor (*AHR*) gene structure and localization of in frame deletion. Double-headed arrow lines identify the regions A–D amplified by PCR; single arrow lines identify primers used to amplify deletion region. Representative PCR for *AHR* exon 10 regions of tumor DNA of patient #5. The leftmost lane contains DNA ladder (1kb plus). Lane 2 shows PCR product of exonic region upstream the variant rs764425858 (PCR#A). Lane 3 shows PCR product of exonic region downstream the variant rs764425858 and upstream the variant rs760349720 (PCR#B). Lane 4 shows PCR product of exonic region downstream variants rs760349720 and rs760349720 and upstream the variant rs778350893 (PCR#C). Lane 5 shows PCR product of exonic region between the variants rs760349720 and rs778350893 (PCR#D). White lines indicate PCR products run in non-contiguous lanes of the same gel. **(B, C)** Representative sequence analysis on cDNA after amplification of *AHR* exon 9 and 10 in healthy pituitary sample **(B)** and tumor samples (patient #5) **(C)**. Left panel shows electropherogram with a focus on the amplified region c.1027-c.1243 (PCR of exon 9 and upstream exon 10, before deletion site; c.1181-c.1243). Right panel shows electropherogram with a focus on the amplified region c.1224-c.1243 (PCR of exon 9 and upstream exon 10, after deletion site (c.1027-c.1579).

To corroborate the presence of a potential deletion associated with this region we performed a series of PCR using pairs of primers designed before and after known insertion and/or deletion variants throughout exon 10 of the *AHR* gene. The primers design was according with the SNP database (Short Genetic Variation;/www.ncbi.nlm.nih.gov) as germline pathogenic mutations: rs764425858 (in-frame deletion HGVS Name: c.1246_1254del; p.Ala416_Asn418del; Position g.17339071_17339079del) rs760349720 (in frame deletion HGVS Name: c.1481_1483del; p.Arg494del; Position g.17339306_17339308del) and rs778350893 (in frame deletion HGVS Name: c.1893_1895del; p.Val632del; Position g.17339718_17339720del). Of note, to date there are no somatic pathogenic deletions upstream of exon 10 reported in COSMIC database (*Catalogue of Somatic Mutations In Cancer)*. We screened specific exonic regions: upstream the variant rs764425858 (PCR#A; intron 9-10 - c.1243); downstream rs764425858 and upstream rs760349720 (PCR#B; c.1260-c.1579); downstream rs760349720 and/or between the variants rs760349720 and rs778350893 (PCR#C-D; c.1497-c.1807 and c.1807-c.2055). Of interest, we observed amplification only before codon 1246 and after codon 1254 (rs764425858), strengthening the evidence of a somatic deletion in our patients (**Figure 2A**) in the 5’ upstream region of exon 10, corresponding to the PCR-binding site of primer used for mutational analysis (chr7:17339632+17340337, c.1239-c.2056) (13). Sequencing data of these samples were then screened to validate the presence of the in frame deletion rs764425858 at somatic level. Results revealed an altered sequence of *AHR* at this level compared to germline DNA (**Figure S4**).

Of interest, we were able to extract RNA from FFPE tumor specimens of nine of our patients and performed mutational analyses on cDNA after amplification of exon 9 and 10 to validate the effect of this deletion at the mRNA level. Sequencing analysis of PCR-amplified cDNA (**Figure 2B, C** and **Figure S5**, **S6****)** revealed: *i*. a correct sequence of exon 9 and 10 up to codon 1246 (**Figure 2C** and **Figure S6**, left panel; amplification region c.1027-c.1243); *ii*. the lack of sequence data due to the absence of the priming site after the deletion region (c.1246-c.1254) (**Figure 2C** and **Figure S6**, right panel; amplification region c.1027-c.1579) in 6 of 9 tumor samples, compared to pituitary normal tissue, used as reference control (**Figure 2B** and **Figure S5**). The lack of specific amplification of these genomic regions in the other three patients might be due to the low amount of RNA obtained.

The above observation is consistent with the hypothesis that GH-secreting pituitary adenoma carries this variant, and that this somatic mutation is likely to be inactivating and therefore potentially affecting pituitary tumor pathophysiology.

### Evaluation of Loss of Heterozygosity

It is known that the germline SNP rs2066853 may have an impact on *AHR* gene structure/function and that this polymorphism is more frequent among acromegaly patients and associated with a worse clinical outcome, biochemical profile and more invasive pituitary tumor. Given the evidence that this SNP is more frequent in heterozygosis and the phenomenon of loss of heterozygosity (LOH) is associated with tumor progression, we assumed that this variant and its LOH might be involved in pituitary tumor pathogenesis.

**Figure 3:**
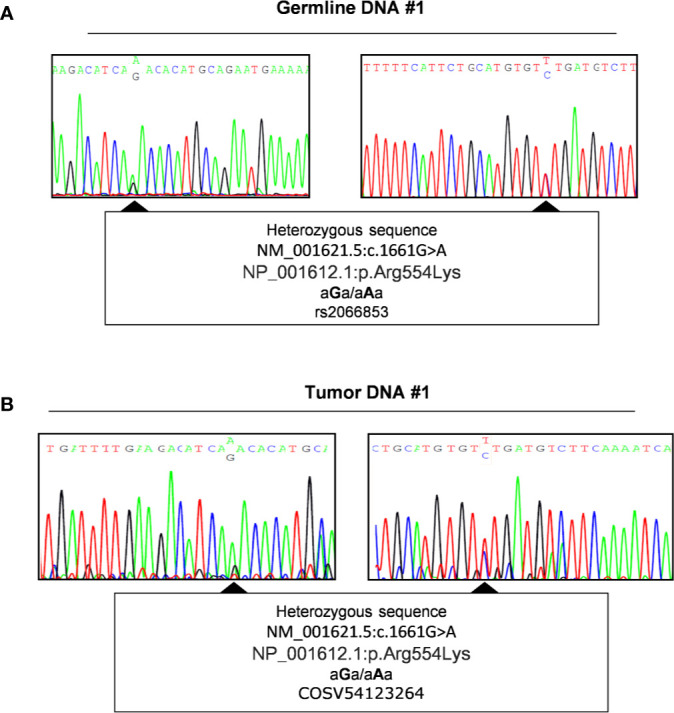
Evaluation of Loss of heterozygosity. Representative sequence analysis of aryl hydrocarbon receptor (*AHR*) exon 10, surrounding region of polymorphism rs2066853, in germline **(A)** and tumor DNA **(B)** of the same patient (#1). The polymorphism rs2066853 (c.1661 G>A) or its correspondent genomic mutation ID COSV54123264 are arrowed in germline **(A)** and tumor DNA **(B)**, respectively. Left and right panels show sequence analysis performed with forward and reverse primers, respectively.

To investigate a potential LOH we performed direct sequencing analysis of the genetic locus of *AHR* in tumor DNA of patients carrying or not this germline variant. We therefore amplified the downstream region of exon 10 including SNP rs2066853 using primers designed after the site of in frame deletion rs764425858 (codon 1254). Data revealed the absence of loss of heterozygosis of this missense variant (Genomic Mutation ID: COSV54123264) in patients carrying the variant rs2066853 at germline context (**Figure 3** and **Figures S7-10**).

## Discussion

AHR is a ligand-activated transcription factor that can bind over 400 different endogenous and exogenous compounds, including several endocrine disrupting chemicals and environmental contaminants (17–19). AHR activation leads to the transactivation of genes encoding phase I and II xenobiotics metabolizing enzymes, thus representing a key factor of the intracellular detoxification systems (20). The AHR has been therefore studied mainly in the context of environmental pollutants processing as transcriptional regulator of genes involved in the metabolism and/or excretion of toxins such as dioxin TCDD (21). Moreover, AHR is directly or indirectly involved in many other biologically relevant processes - *via* the so-called non-canonical pathways – such as cell cycle regulation and cell contact inhibition, potentially contributing to tumorigenesis (22–24). A limited number of studies has focused on the impact of AHR signaling in PA. Jaffrain-Rea in 2009 reported that the AHR and its partner AIP were downregulated in aggressive somatotropinomas as compared to non-invasive adenomas suggesting their involvement in the acquisition of an aggressive phenotype (25). Subsequently, several groups focused their attention on the uncovered link between AHR and/or AIP expression and the molecular mechanisms underlying pituitary adenoma pathogenesis. AIP, a co-chaperone protein that acts as a tumor suppressor in pituitary cells (26), is critical for AHR stabilization and function. AIP gene germline mutations have been found in young patients with familial or apparently sporadic aggressive pituitary adenomas, mostly secreting GH and/or PRL, and less responsive to conventional medical treatments (27). Moreover, although it is still a matter of debate, AIP mutations also affect the AHR signaling, since they can alter the AHR and phosphodiesterase (PDE)4A5 interaction and consequently the cAMP pathway, affecting pituitary function and tumorigenesis (22, 28). Several AHR gene polymorphisms have been described, mostly in exon 10, with some of them associated with an altered induction of CYP1A1 and CYP1A2 activity in response to specific ligands (29).

Our group showed that the germline single nucleotide polymorphism (SNP) rs2066853 of the AHR encoding gene is more frequent in acromegaly patients as compared to sex- and age-matched healthy subjects and is associated with increased pituitary adenoma invasiveness and secondary neoplasm’s risk (13). Of interest, in 2016 a study revealed that this SNP, causing an arginine to lysine substitution in the TAD domain at codon 554 (R554K), may affect AHR protein structure and function (30). To date only germline pathogenic variants of AHR and AIP have been described and, beyond GNAS gene somatic mutations, no obvious driver somatic alterations have been so far identified in GH-secreting pituitary adenoma (31, 32). Interestingly, all *AHR* SNPs described so far reside in exon 10, encoding the TAD, and are associated with other neoplasms, including gliomas (13, 14, 33).

The present study describes for the first time a recurrent somatic sequence alteration in exon 10 of *AHR* gene associated with GH-secreting macroadenoma. Sequence analysis of DNA and RNA from somatotropinoma histological samples derived from patients carrying or not the germline rs2066853 *AHR* variant revealed an in frame deletion on *AHR* coding sequence c.1246-1254 as compared to patients’ matched germline DNA and pituitary normal tissue. It is a somatic deletion exclusively in the tumor tissue. Of interest, this sequence variant, known as germinal polymorphism rs764425858 (GRCh38/hg38, Chr7:17339071-17339079) with a highest minor allele frequency (MAF) <0.01% (EVA_EXAC, GNOMAD, Genomes Project phase III and Ensembl) is located within a regulatory region with structural feature resembling an enhancer (ENSR00001121919). Specifically, Ensembl and Encode databases report a specific interaction between RNA polymerase and transcription factors, such as C-Fos, CREB, and EZH2, capable to control transcription in a cell type or tissue-dependent manner. This might imply that this AHR somatic deletion could interfere with co-regulators and transcription factors potentially impairing the AHR function. Moreover, the somatic alteration is located within TAD domain at the C-terminal region of AHR. The lack of coding sequence in this region might affect protein structure. In light of these considerations we speculated that in pituitary tumors of acromegaly patients with or without AHR/AIP germline genetic variants, this novel somatic deletion of AHR exon 10 (c.1246-1254) could contribute to alter the AHR pathway and favor tumor development. The limitations of this study are the relatively small number of cases, which is mainly due to several factors: *i*) the disease is rare; a limited number of donors were available, *ii*) a single center has carried out sample collection, *iii*) tumors in the study are all somatotropinomas. Besides, frequently surgical samples did not contain sufficient material to allow efficient yields for ‘pure’ pituitary tumor DNA and RNA extraction and analysis. Because of the limitations listed above, our study was performed on a restricted number of samples. Hence, our findings, although consistent, must be considered purely indicative.

In conclusion, this is the first demonstration of a recurrent somatic deletion in the exon 10 of the *AHR* gene in GH-secreting tumors, whose functional impact however needs to be clarified.

## Data Availability Statement

The original sequence data presented in the study are included in the article as **Supplementary Material** and will be publicly available in the “ClinVar” database SCV001433605.

## Ethics Statement

The studies involving human participants were reviewed and approved by Ethics Committee University of Messina, Italy. The patients/participants provided their written informed consent to participate in this study.

## Author Contributions

AR, FF, SC, and AF conceived and designed the research. FS, MR, DR, and VB enrolled patients and managed clinical, biochemical, and histological data. CC revised data analysis and manuscript text. AR carried out experiments and data analysis. CG, SC, and AP contributed reagents, materials, and analysis tools. AR, FF, SC, and AF carried out data analysis and interpreted the results. AR and AF drafted the manuscript. FF and SC revised the manuscript. All the authors contributed critical discussion and approved the final version of the manuscript. All authors contributed to the article and approved the submitted version.

## Funding

The present study was supported by the Italian Ministry of Education, University and Research (MIUR) (PRIN2017S55RXB) to SC and AF. This study was partially funded by FISR-MIUR “Centro Internazionale di Studi Avanzati su Ambiente, ecosistema e Salute umana” CISAS (B62F15001070005).

## Conflict of Interest

The authors declare that the research was conducted in the absence of any commercial or financial relationships that could be construed as a potential conflict of interest.
