# Supplementary material for: Somatic Deletion in Exon 10 of Aryl Hydrocarbon Receptor Gene in Human GH-Secreting Pituitary Tumors
Source: Front Endocrinol (Lausanne). 2020 Nov 12;11:591039. doi: 10.3389/fendo.2020.591039 (PMC7689685; doi:10.3389/fendo.2020.591039)
Supplement: Supplementary file 1 [file Presentation_1.pptx]

## Slide 1
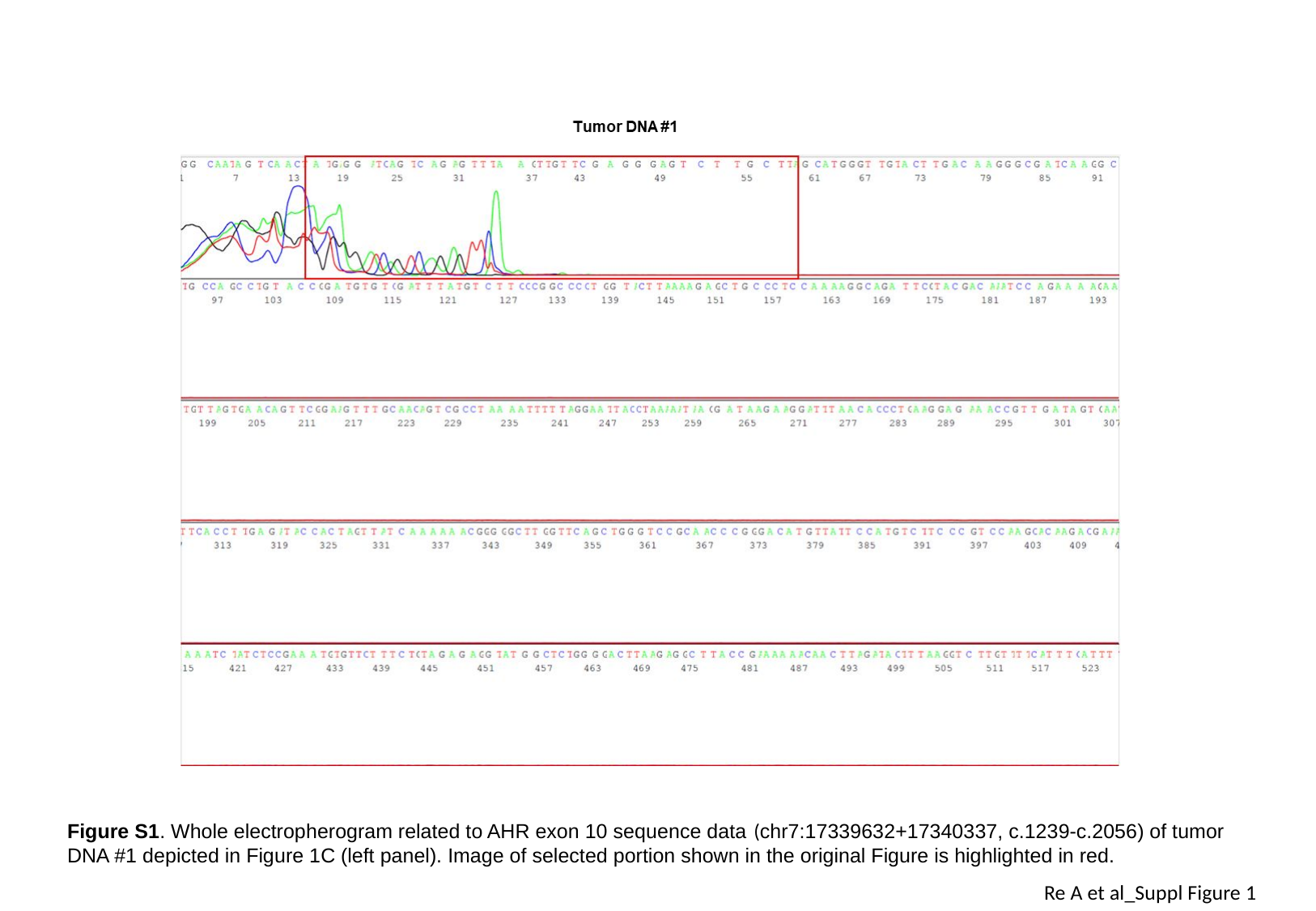

Figure S1. Whole electropherogram related to AHR exon 10 sequence data (chr7:17339632+17340337, c.1239-c.2056) of tumor DNA #1 depicted in Figure 1C (left panel). Image of selected portion shown in the original Figure is highlighted in red.
Re A et al_Suppl Figure 1

## Slide 2
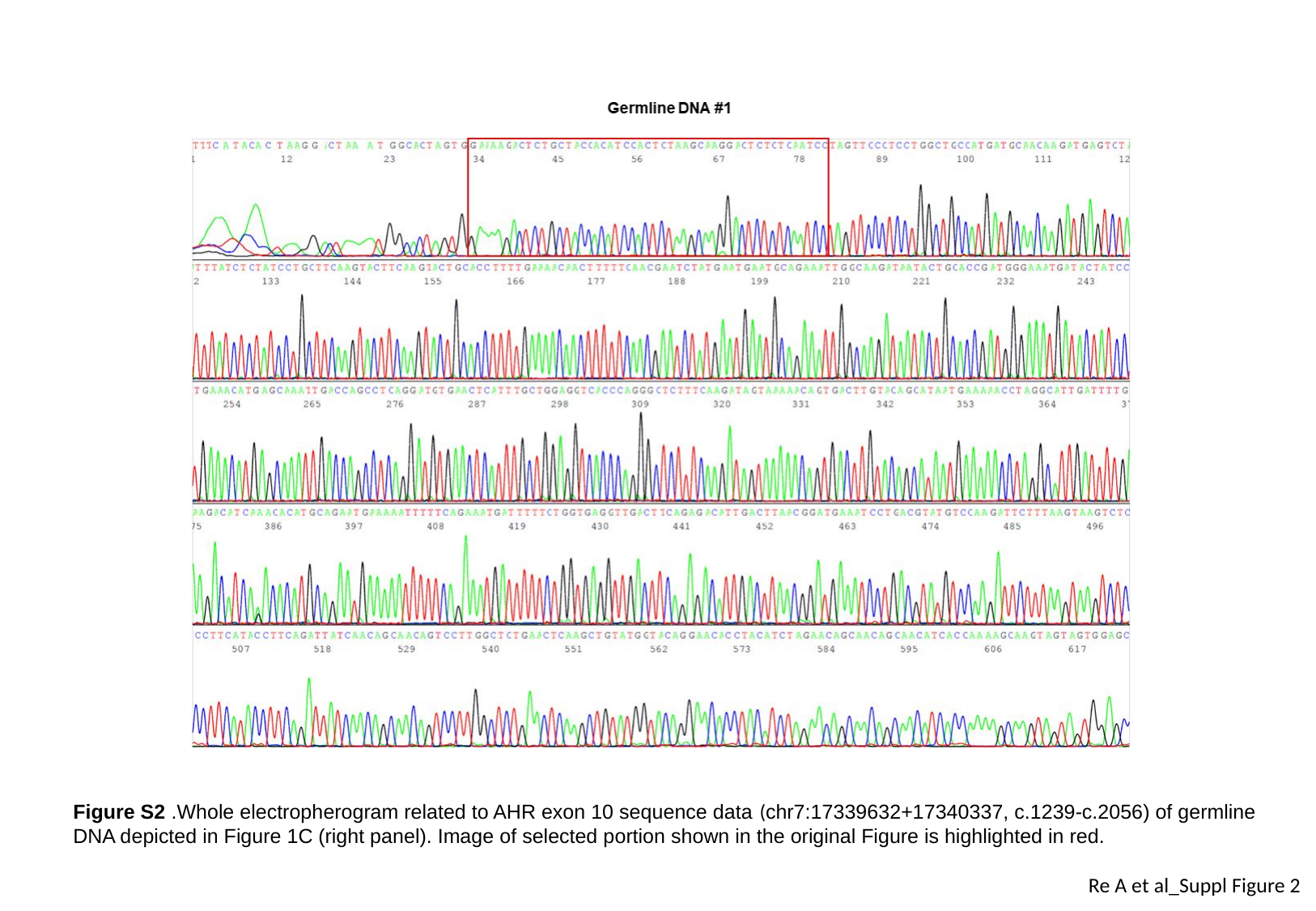

Figure S2 .Whole electropherogram related to AHR exon 10 sequence data (chr7:17339632+17340337, c.1239-c.2056) of germline DNA depicted in Figure 1C (right panel). Image of selected portion shown in the original Figure is highlighted in red.
Re A et al_Suppl Figure 2

## Slide 3
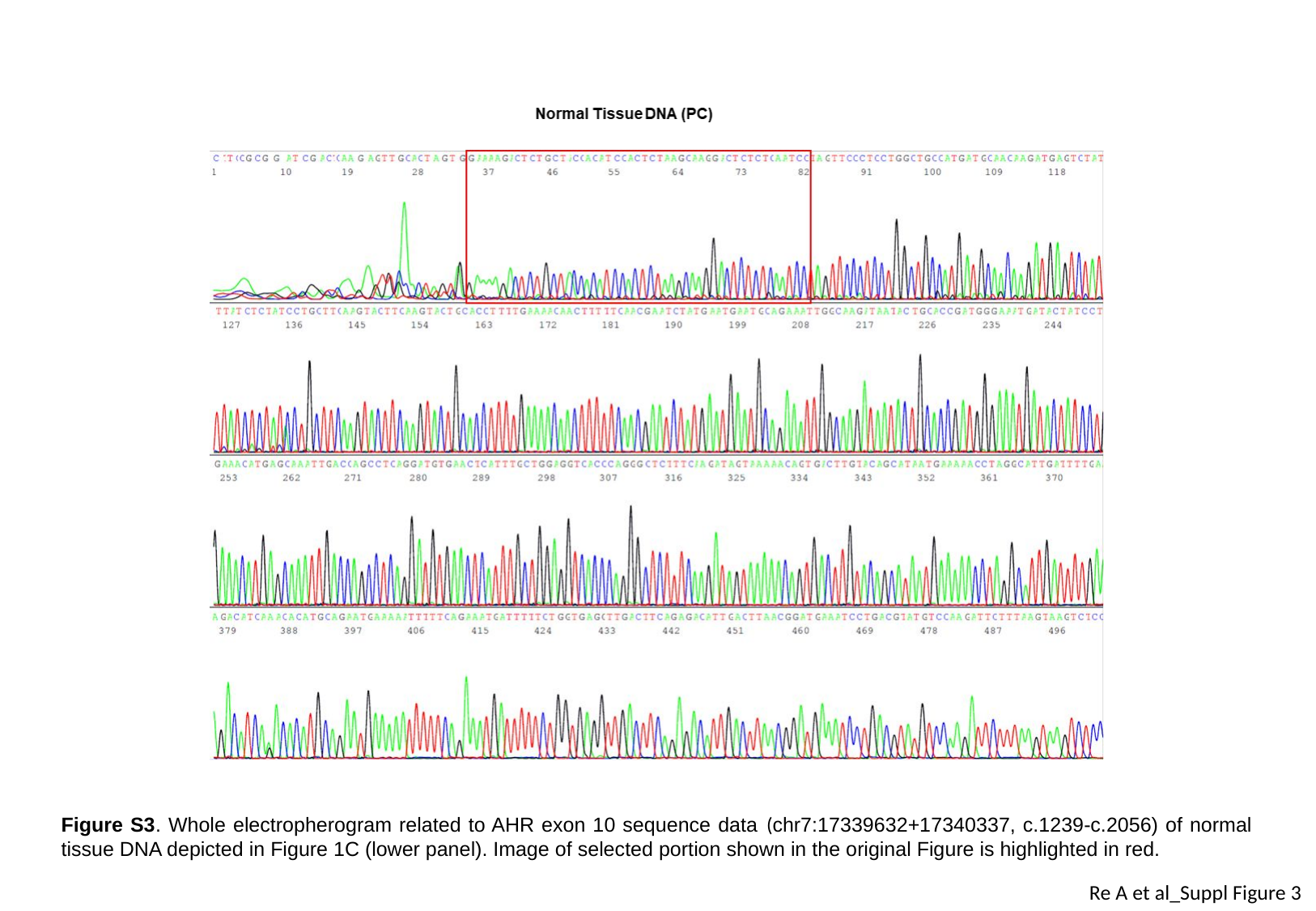

Figure S3. Whole electropherogram related to AHR exon 10 sequence data (chr7:17339632+17340337, c.1239-c.2056) of normal tissue DNA depicted in Figure 1C (lower panel). Image of selected portion shown in the original Figure is highlighted in red.
Re A et al_Suppl Figure 3

## Slide 4
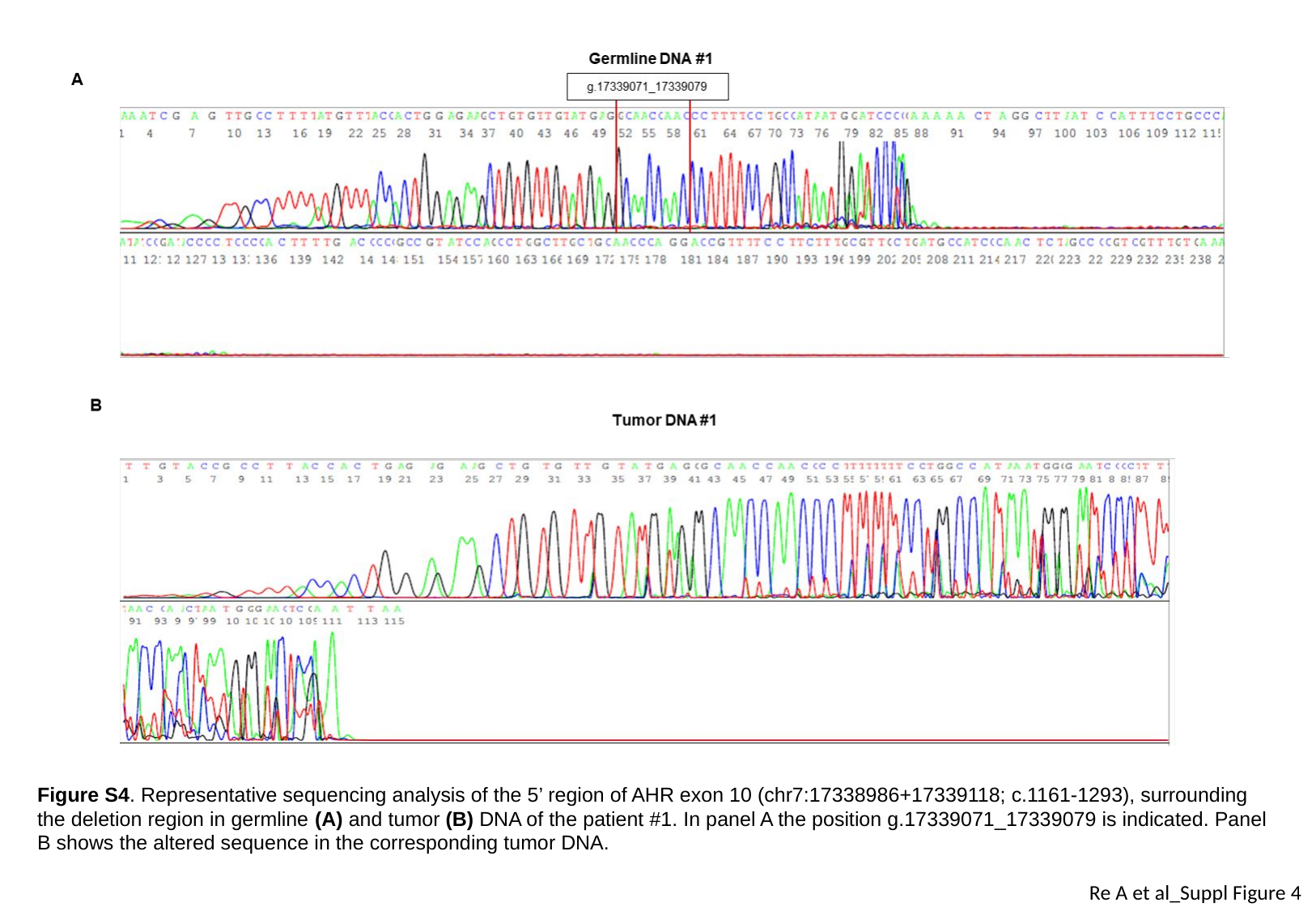

Figure S4. Representative sequencing analysis of the 5’ region of AHR exon 10 (chr7:17338986+17339118; c.1161-1293), surrounding the deletion region in germline (A) and tumor (B) DNA of the patient #1. In panel A the position g.17339071_17339079 is indicated. Panel B shows the altered sequence in the corresponding tumor DNA.
Re A et al_Suppl Figure 4

## Slide 5
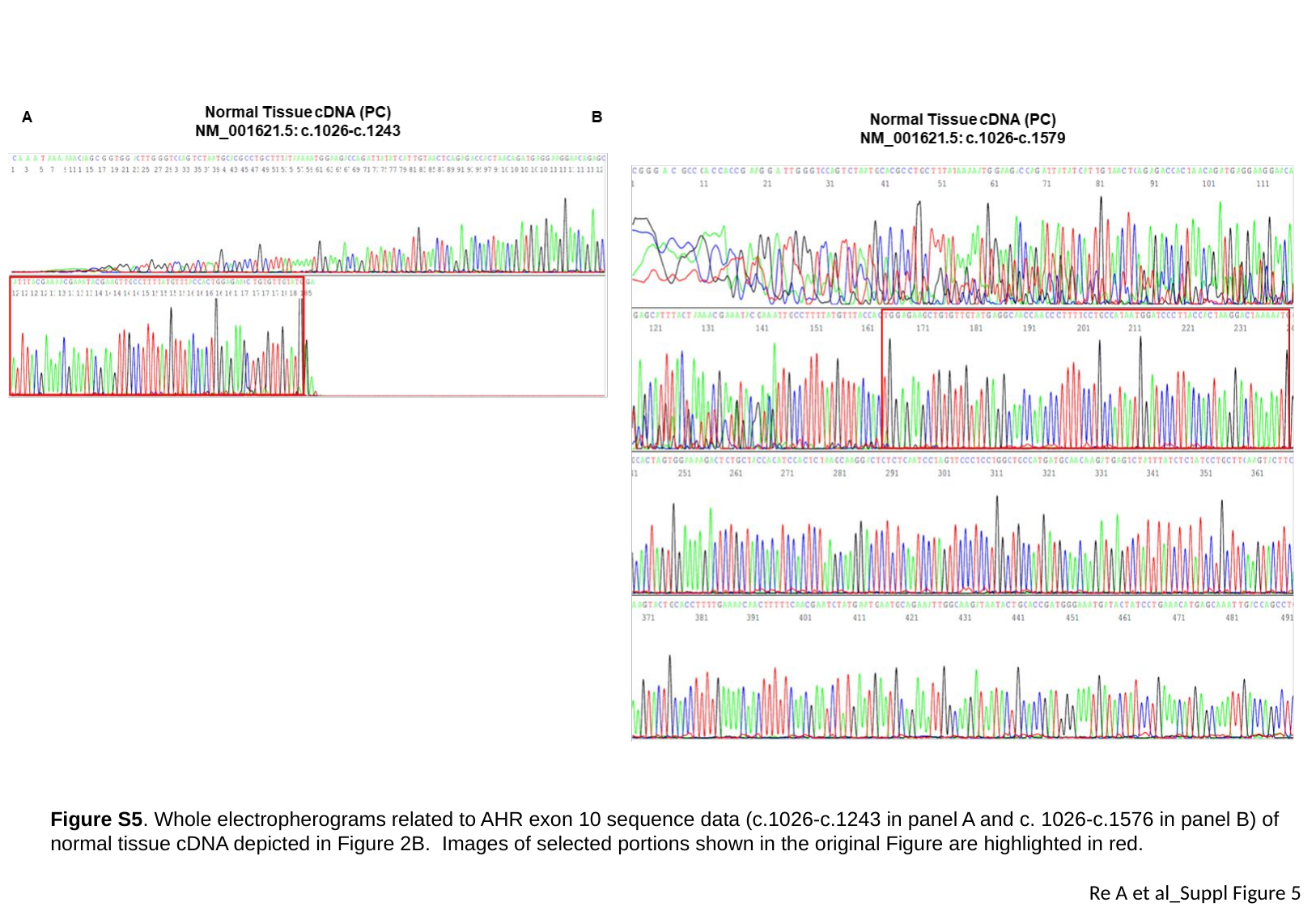

Figure S5. Whole electropherograms related to AHR exon 10 sequence data (c.1026-c.1243 in panel A and c. 1026-c.1576 in panel B) of normal tissue cDNA depicted in Figure 2B. Images of selected portions shown in the original Figure are highlighted in red.
Re A et al_Suppl Figure 5

## Slide 6
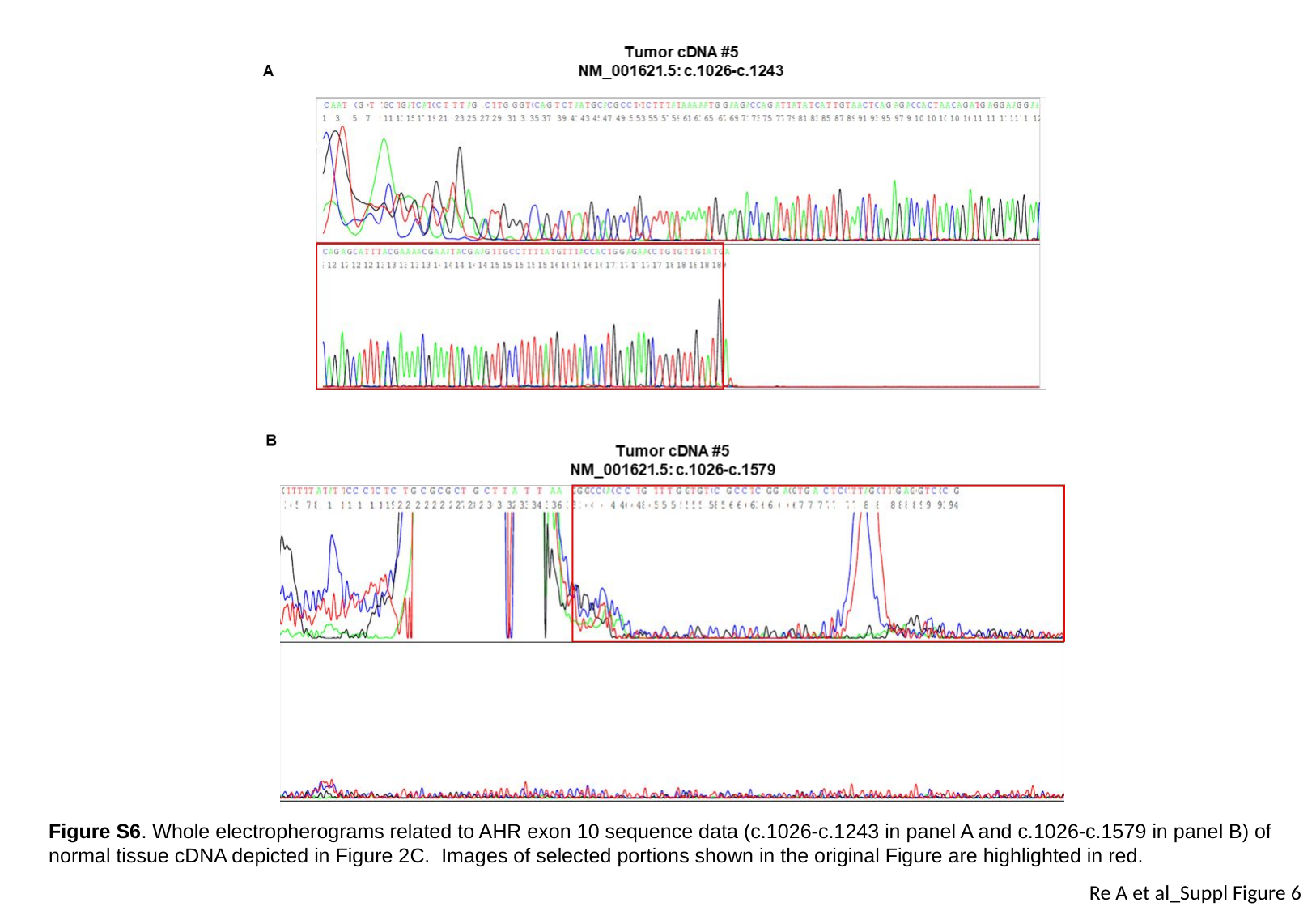

Figure S6. Whole electropherograms related to AHR exon 10 sequence data (c.1026-c.1243 in panel A and c.1026-c.1579 in panel B) of normal tissue cDNA depicted in Figure 2C. Images of selected portions shown in the original Figure are highlighted in red.
Re A et al_Suppl Figure 6

## Slide 7
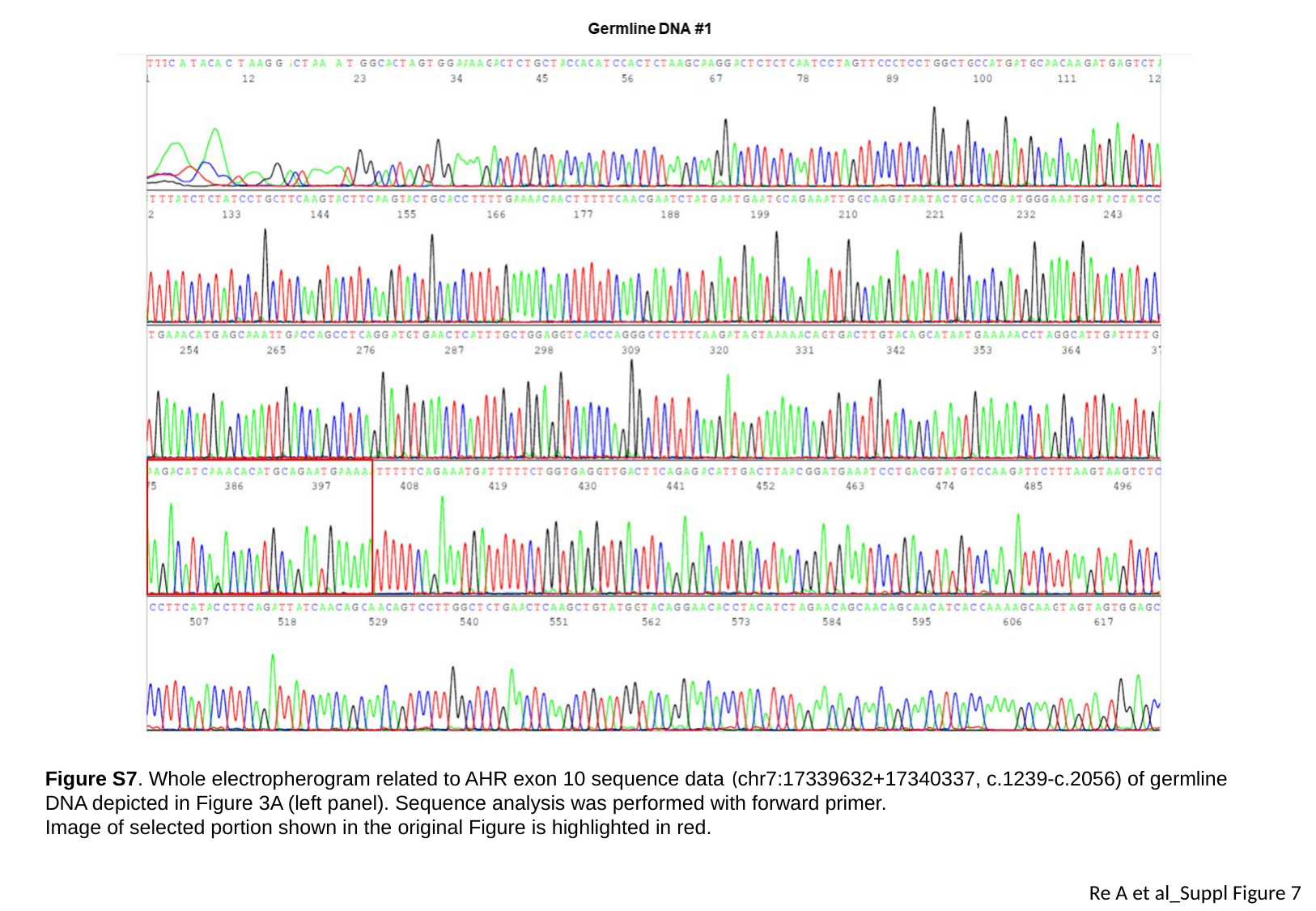

Figure S7. Whole electropherogram related to AHR exon 10 sequence data (chr7:17339632+17340337, c.1239-c.2056) of germline DNA depicted in Figure 3A (left panel). Sequence analysis was performed with forward primer.
Image of selected portion shown in the original Figure is highlighted in red.
Re A et al_Suppl Figure 7

## Slide 8
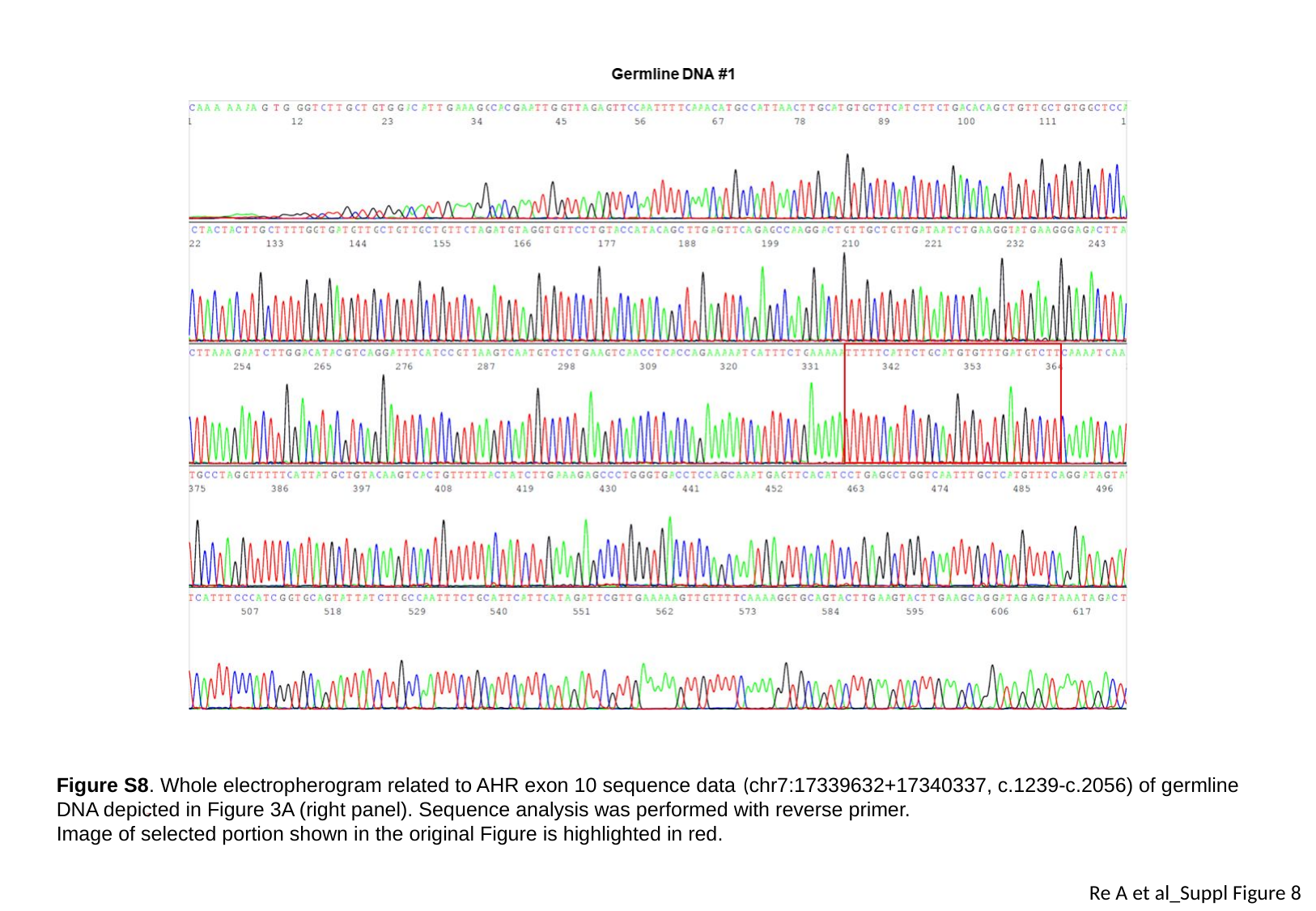

Figure S8. Whole electropherogram related to AHR exon 10 sequence data (chr7:17339632+17340337, c.1239-c.2056) of germline DNA depicted in Figure 3A (right panel). Sequence analysis was performed with reverse primer.
Image of selected portion shown in the original Figure is highlighted in red.
Re A et al_Suppl Figure 8

## Slide 9
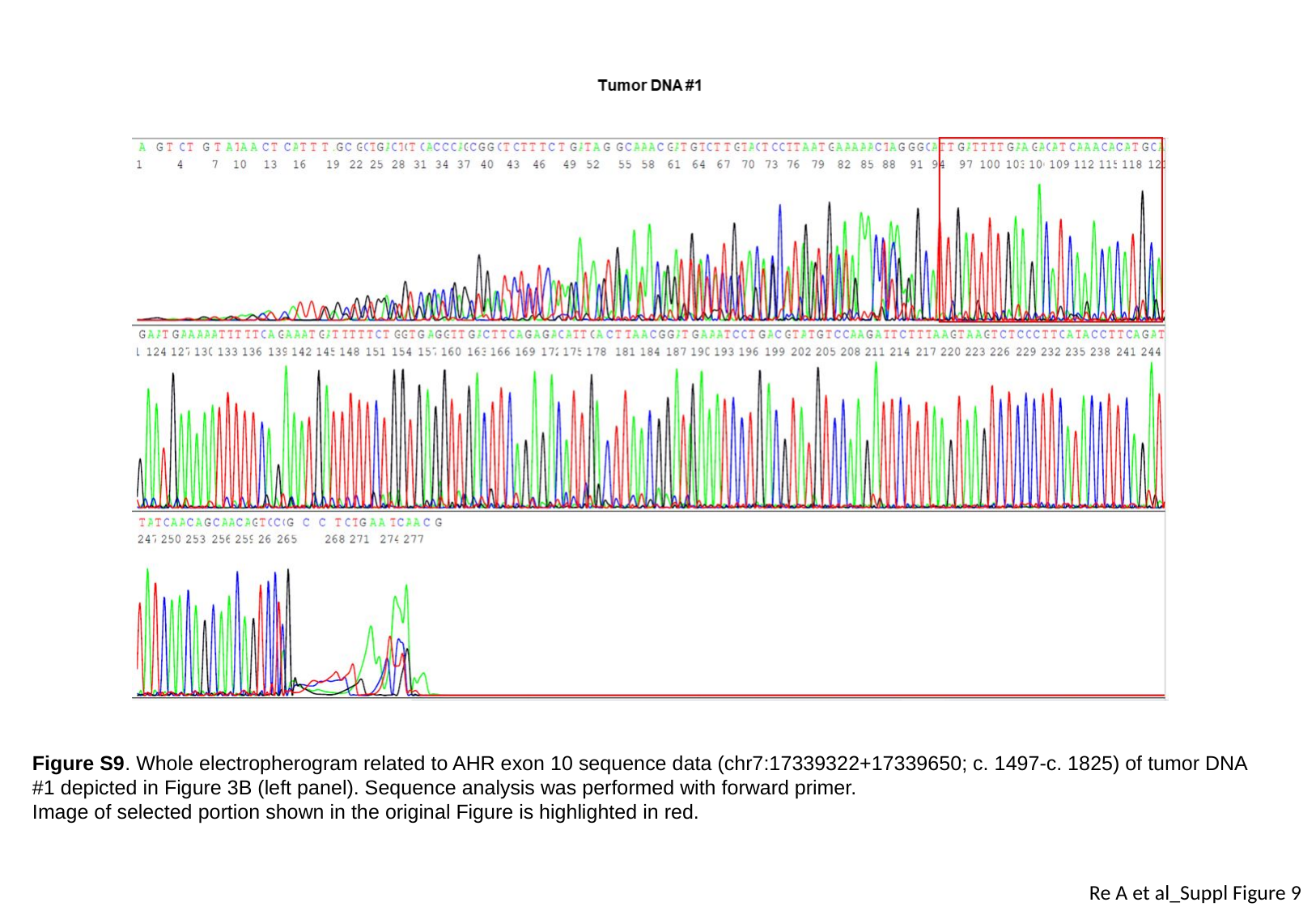

Figure S9. Whole electropherogram related to AHR exon 10 sequence data (chr7:17339322+17339650; c. 1497-c. 1825) of tumor DNA #1 depicted in Figure 3B (left panel). Sequence analysis was performed with forward primer.
Image of selected portion shown in the original Figure is highlighted in red.
Re A et al_Suppl Figure 9

## Slide 10
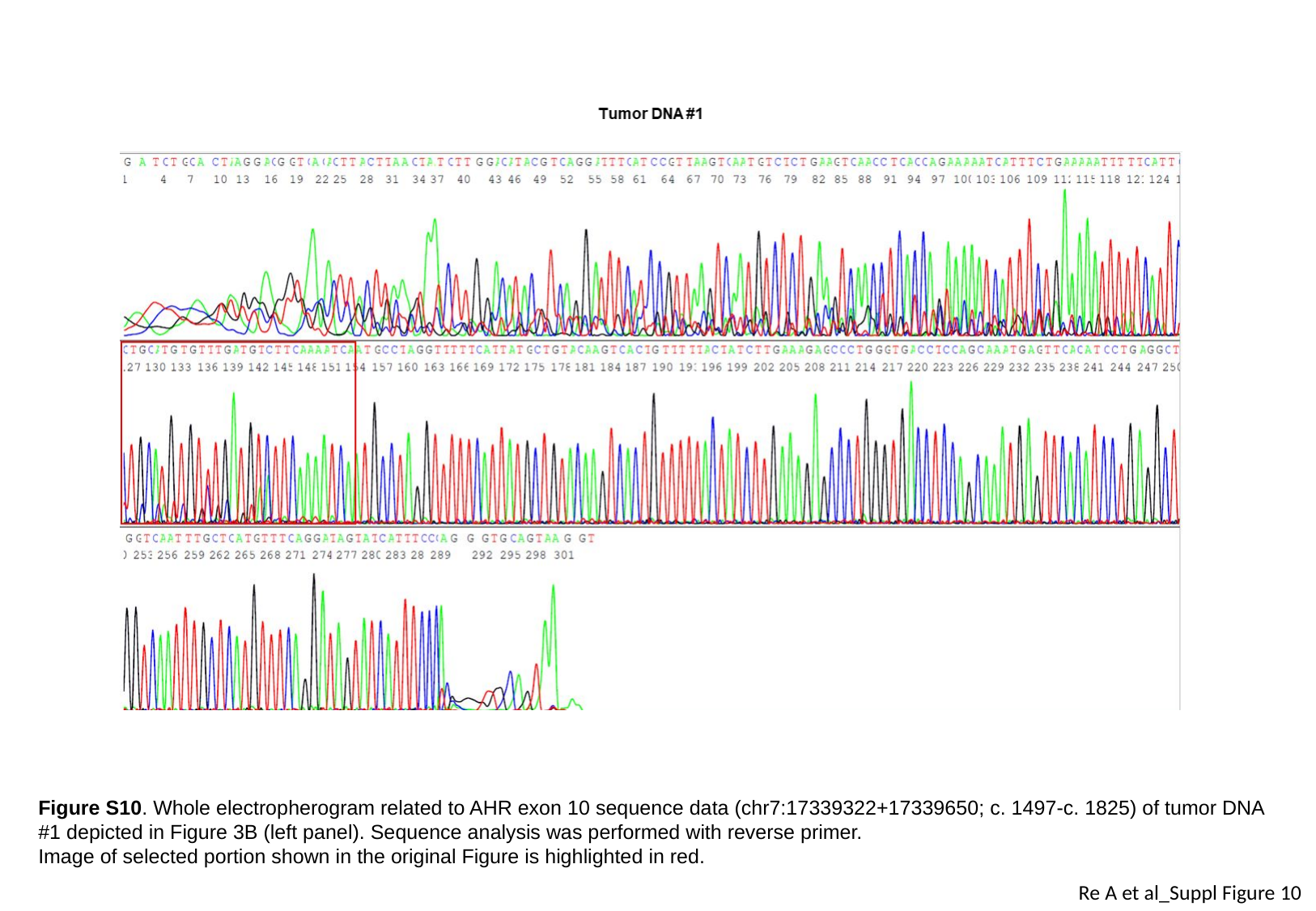

Figure S10. Whole electropherogram related to AHR exon 10 sequence data (chr7:17339322+17339650; c. 1497-c. 1825) of tumor DNA #1 depicted in Figure 3B (left panel). Sequence analysis was performed with reverse primer.
Image of selected portion shown in the original Figure is highlighted in red.
Re A et al_Suppl Figure 10
